# An Anthracene-Thiolate-Ligated Ruthenium Complex: Computational Insights into Z-Stereoselective Cross Metathesis

**DOI:** 10.1021/acs.jpca.3c05021

**Published:** 2023-11-02

**Authors:** Juan Pablo Martínez, Bartosz Trzaskowski

**Affiliations:** Centre of New Technologies, University of Warsaw, Banacha 2C, 02-097 Warszawa, Poland

## Abstract

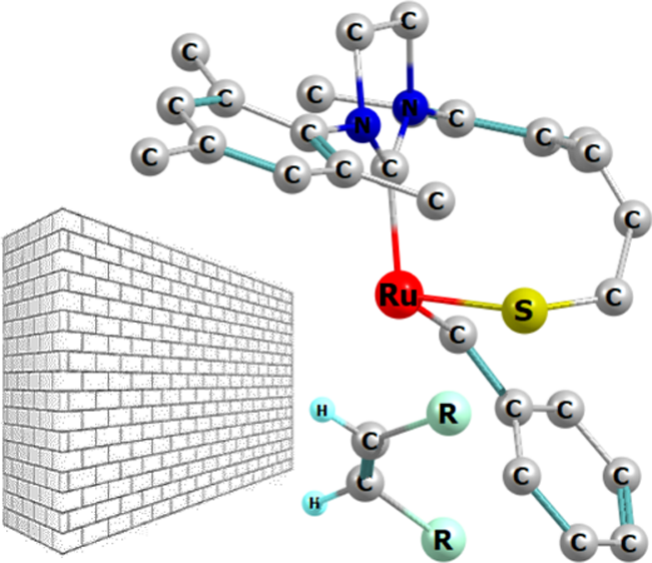

Stereoselective control of the cross metathesis of olefins is a crucial aspect of synthetic procedures. In this study, we utilized density functional theory methods to calculate thermodynamic and kinetic descriptors to explore the stereoselectivity of cross metathesis between allylbenzene and 2-butene-1,4-diyl diacetate. A ruthenium-based complex, characterized primarily by an anthracene-9-thiolate ligand, was designed in silico to completely restrict the E conformation of olefins through a bottom-bound mechanism. Our investigation of the kinetics of all feasible propagation routes demonstrated that Z-stereoisomers of metathesis products can be synthesized with an energy cost of only 13 kcal/mol. As a result, we encourage further research into the synthetic strategies outlined in this work.

## 1 Introduction

**Figure 1:**
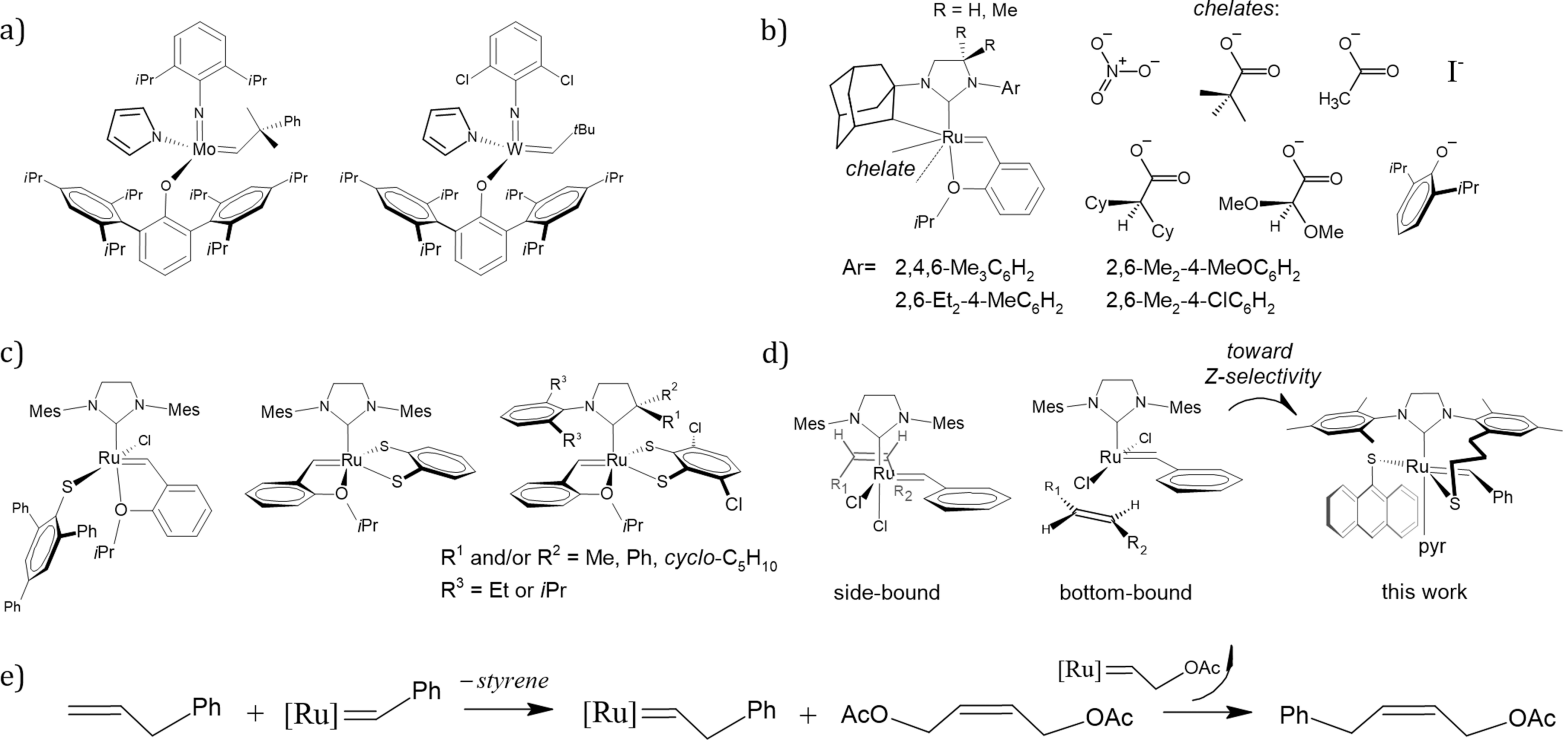
Selected Z-stereoselective catalysts: (a) early Mo- and W-based complexes, (b) set of catalysts designed by Grubbs, and (c) Ru-based complexes ligated to thiolates. (d) Comparison between the side-bound and bottom-bound conformations as a strategy in the design of Z-selective catalysts. (e) Cross metathesis studied in this work.

Historically, one of the main challenges in cross metathesis (CM) was the control of the stereoselectivity of the reaction.^1^ For nonstereoselective catalysts, metathesis products typically consist of a mixture of E and Z isomers, creating operational difficulties due to the complicated and costly nature of component separation.^2^ In 2009, Schrock et al. synthesized the first Z-selective olefin metathesis catalysts based on Mo^3,4^ and W^5^ complexes (see Figure 1). These catalysts were able to achieve yields above 50% with a Z isomer ratio greater than 98%. At that juncture, the field of alkylidene chemistry involving Mo and W had advanced substantially, resulting in the availability of numerous exceptionally active and tunable catalysts.^6^ In addition, over the past decade, significant progress has been made in the development of Z-selective Mo- and W-based catalysts for CM, which have provided new opportunities for the synthesis of Z-olefins with high selectivity and efficiency.^7^ These include, for example, a series of halogenated Mo-alkylidene complexes that were reported as exceptionally effective catalysts for olefin CM reactions, resulting in the production of acyclic 1,2-disubstituted Z-alkenyl halides with ratios Z:E of 93:7 or higher.^8^ Monoaryloxide chloride Mo-based catalysts also exhibited Z-selective CM reactions involving a series of olefins and Z-1,1,1,4,4,4-hexafluoro-2-butene in benzene at 22 °C.^9^ Furthermore, in the same context of the Mo-catalyzed CM framework, notable advances in the synthesis of Z-trisubstituted alkenes have been recently achieved by Hoveyda et al.^10−12^

In the case of Z-selective Ru-based catalysts, a series of functional-group-tolerant systems were reported by Grubbs et al. since 2011.^13,14^ These catalysts are composed of an *N*-heterocyclic carbene (NHC) ligand and were derived from the intramolecular carboxylate-driven C–H bond insertion of an *N*-bound substituent (e.g., adamantyl in Figure 1b).^15−18^ Nitrato-substituted catalysts were found to be more stable and efficient for CM.^19^ In 2013, Hoveyda et al. used a similar approach to synthesize a Z-selective catalyst, where the replacement of chlorides with a catechodithiolate ligand favored the formation of the Z isomer in ring-opening metathesis polymerization and ring-opening/cross-metathesis.^20^ In the same year, Jensen et al. achieved Z-selective CM by substituting one chloride of a Ru-based catalyst complex with the bulky 2,4,6-triphenylbenzenethiolate (see Figure 1c).^21^ This substitution resulted in Z selectivity of up to 96% in the metathesis homocoupling of terminal olefins. Continued investigations in Jensen’s group resulted in additional phosphine-based Z-selective complexes.^22^ In 2015, within Ru-based catalysts, Hoveyda et al. presented a Ru-disulfide complex to catalyze the synthesis of acyclic Z allylic alcohols via CM transformations, with yields of up to 80% and Z:E ratios as high as 98:2.^23^ In 2018, Mauduit et al. synthesized a variation of the adamantyl-NHC catalysts, which incorporated *N*-Dipp (*N*-2,6-diisopropylphenyl) and *N*-adamantyl substituents in the nonsaturated NHC ligand.^24^ This catalyst exhibited exceptional catalytic efficacy in both self- and CM reactions, even at a low catalyst loading, yielding internal olefins with remarkable conversion rates and high Z-selectivity exceeding 99%. Later in 2023, within the Ru-based framework, Mauduit’s group synthesized a series of 16 Z-selective catalysts, and in the case of a catechodithiolate complex, it resulted in a remarkable performance in the Z-selective (>98%) asymmetric ring-opening CM of exonorbornenes.^25^ Furthermore, the first Z-stereoselective catechodithiolate-based ruthenium complexes containing cyclic(alkyl)(amino)carbene (CAAC) ligands were recently reported by Bertrand and co-workers (see Figure 1c).^26^ Moderate to good yields and high Z-selectivity (>98%) were obtained in various metathesis transformations, including CM.

Grubbs et al. in collaboration with Houk’s group provided a rationale for the design of Z-stereoselective catalysts by distinguishing between two possible metathesis mechanisms, bottom-bound^27^ and side-bound^28^ (see Figure 1d).^29^ They presented evidence to support the notion that the side-bound mechanism leads to improved d-orbital back-donation and van der Waals interactions between the catalyst and the Z isomer of olefin in transition states, and the steric compression of the olefin substituents is reduced. A possible explanation for the Z-selectivity observed in catechodithiolate-based catalysts may be rationalized in terms of the side-bound mechanism as reported by Houk et al.^29^ Sterically demanding ligands are known to stabilize the 14-electron Ru-activated catalyst^30^ and prevent the formation of the E isomer.^31,32^ However, Z-selective catalysts that incorporate monodentate chelates (e.g., I^–^, (2,4,6-Ph_3_)Ph-S^–^, or Dipp-O^–^) that allow bottom-bound pathways have also been developed. As a result, the question of whether the Z-stereoselectivity arises entirely from the side-bound mechanism is a primary focus in the current study.

Recent developments in Z-stereoselective olefin metathesis have led to specific applications such as continuous-flow synthesis of natural products, including pheromones and macrocyclic odorant molecules.^33^ Inspired by the current state of Z-selective olefin metathesis, we aimed to investigate by means of quantum-chemical methods the development of an even more efficient catalyst for stereoselective CM reactions. To achieve this goal, we incorporated a polycyclic aromatic-carbon (anthracene) thiolate ligand to exclusively induce the formation of Z olefins. It may be argued that this framework replicates the design principles of Jensen’s catalyst,^21^ in which 2,4,6-triphenylbenzenethiolate was incorporated as a ligand that induces the selective formation of Z olefins. Although similarities exist, ligands such as anthracenes or extended polycyclic rings cannot be spatially reoriented so that the formation of E olefins is prohibitive. Therefore, our current research is motivated by the observation that E olefins could still be detected in catalysts with improved Z-selectivity, which were synthesized through the incorporation of Z-inducing ligands such as thiolates,^22^ sulfonates, and phosphates.^34^

Accordingly, we chose a model reaction from a set of six metathesis transformations proposed by Grubbs et al. to characterize newly synthesized catalysts,^31,35^ specifically the CM of allylbenzene (**A**) and 2-butene-1,4-diyl diacetate (**B**) to produce 4-phenyl-2-buten-1-yl acetate (**P**) (see Figure 1e). The objective of our work was to conduct a thorough analysis of the thermodynamic and kinetic factors that determine the formation of the Z isomer of the products as well as to elucidate key strategies to encourage further experimental assessment. In this context, several researchers have previously documented predictive and descriptive catalyses through studies based on chemical calculations and reactivity models, some of them validated by experimental investigations.^36−40^

## 2 Computational Details

Density functional theory (DFT) calculations were performed using the quantum-chemistry code Jaguar version 11.2.^41^ Becke’s three-parameter functional combined with the Lee–Yang–Parr correlation functional (B3LYP) was employed to optimize the geometries.^42,43^ During the geometry optimization process, the dispersion energy corrections developed by Grimme et al. (D3) were incorporated.^44^ The electronic configuration of the molecular systems was described using the Gaussian 6-31G** basis set, which includes polarization functions for the H, C, N, O, and S atoms. In the case of ruthenium, the small-core, quasi-relativistic effective core potential developed at Los Alamos National Laboratory (LA),^45^ along with an associated double ζ plus polarization basis set, was used (standard LACVP** keyword in the Jaguar code). Analytical frequency calculations were performed for all localized stationary points at the B3LYP-D3/6-31G**∼LACVP** level of theory. Transition states and connecting local minima were located via linear-transit calculations by using the same DFT method. Electronic energies were obtained from the single-point M06-D3 calculations^46^ that included a triple ζ basis set plus polarization and diffuse functions, 6-311G++(d,p) (for Ru: standard LACV3P++** keyword in Jaguar). The polarizable continuum model (PCM)^47,48^ was employed to account for solvent effects, with dichloromethane as the solvent. Gibbs free energies were derived from the corrected electronic energies at the (PCM: CH_2_Cl_2_) M06-D3/6-311G++**∼LACV3P++** // B3LYP-D3/6-31G**∼LACVP** level of theory. Corrections for zero-point energy, thermal contributions to internal energy, and entropy term were calculated from vibrational frequencies at 298.15 K, assuming an ideal gas under standard conditions. Standard convergence criteria and a fine grid for DFT calculations were utilized in all cases. It can be argued that the M06 functional already incorporates medium-range dispersion, leading to overestimation of dispersion contributions due to double counting of these effects in M06-D3.^49^ However, the inclusion of D3 corrections to M06 was demonstrated to enhance the results for catalyzed olefin metathesis, particularly in treating weak interactions.^50^ Furthermore, the computational approach we chose has previously been benchmarked against experimental data.^51,52^

## 3 Results and Discussion

The primary aim of our current work is to demonstrate, via quantum-chemical calculations, the feasibility of designing Z-stereoselective catalysts using the concept of the bottom-bound mechanism. To achieve this goal, we have proposed a Ru-based complex that contains an anionic ligand, anthracene-9-thiolate, which can induce the formation of the Z isomer of olefins through a bottom-bound mechanism. The NHC ligands are constituted of 2,4,6-trimethylphenyl (mesityl) and 2,4-dimethyl-6-(propylthiolate)phenyl groups, where the thiolate group is coordinated with Ru. In this regard, the alkylthiolate moiety was extended until the NHC plane reached a perpendicular alignment with the S–Ru–S axis, resulting in propyl as the alkyl chain. In the context of polycyclic aromatic hydrocarbons, Grela et al. previously synthesized Ru-based catalysts characterized by either a thiophene-based^53^ or a 10-phenyl-9-phenanthryl^54^ ligand bonded to an unsymmetrical NHC moiety. In fact, in the case of Hoveyda–Grubbs complexes bearing *N*-(9-alkylfluorenyl)imidazol-2-ylidene ligands (alkyl: CH_3_, C_2_H_5_, or C_6_H_5_), they conducted thorough investigations of olefin metathesis reactions, with a specific focus on achieving Z-selectivity with Z/E ratios of up to 94/6.^55^ Furthermore, Ru-based catalysts incorporating sulfurated chelates have also been reported by Lemcoff et al.^56−58^ Therefore, we have formulated a possible synthesis route to the precatalyst in question (details in Figure S1 in the Supporting Information), which involves the preparation of thioether-imidazolium chlorides,^59^ anilines,^60,61^ and modifications to the Grubbs first-generation catalyst.^34,62^

### 3.1 The Initiation Route

**Figure 2:**
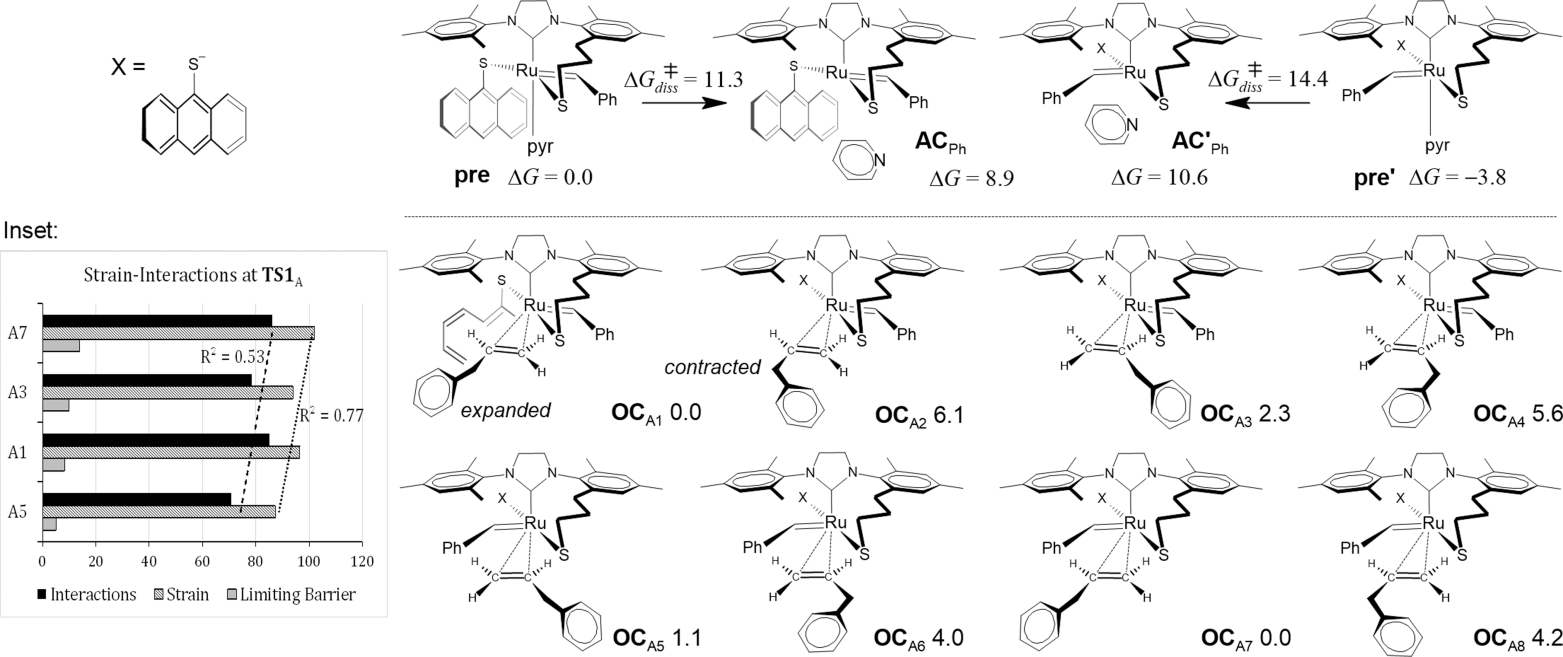
(Top) Gibbs free energy changes for catalyst activation through the dissociation of pyridine. (Bottom) Conformers of the activated catalyst coordinated to allylbenzene (**OC**_A_) and Gibbs free energy comparisons relative to **OC**_A1_. Inset: The activation-strain model for the 2,2-cycloaddtion transition state (**TS1**_A_). All energies are given in kcal/mol.

The catalytic cycle commences with the generation of an active catalyst, **AC**_Ph_, as a result of the dissociation of pyridine from **pre**. Two distinct conformations **pre** and **pre′** are presented in Figure 2, which are differentiated by the orientation of benzylidene relative to the propyl-thiolate of NHC. The Ru-pyridine bond dissociation reported in Figure S2 in the Supporting Information reveals a flat potential (electronic) energy surface that reaches maxima of 16 and 21 kcal/mol for **pre** and **pre′**, respectively. Nonetheless, for the localized stationary points, the thermal contributions resulted in a Gibbs activation energy of <15 kcal/mol, as shown in Figure 2. As a result, activation of the catalyst is expected to take place under mild conditions at room temperature. The subsequent step involves olefin coordination (**OC**), and given our interest in synthesizing 4-phenyl-2-buten-1-yl acetate **P** through a productive metathesis reaction, there exist two possible sequences of the cycle: allylbenzene **A** association followed by propagation with 2-butene-1,4-diyl diacetate **B**, as illustrated in Figure 1e, or vice versa. Nevertheless, **AC**_Ph_ is expected to exhibit a preference for reacting with allylbenzene initially, owing to the greater reactivity of less substituted alkenes such as olefin **A**,^63^ as confirmed in our earlier work.^64^ As a result, we focus only on the initiation route through **A**.

Our analysis and energy comparisons were conducted starting from the olefin coordination intermediate **OC**_A_. We identified eight distinct configurations for the coordination of **A** with the active catalyst **AC**_Ph_ (or **AC′**_Ph_ in which benzylidene is *trans* to propyl-thiolate), as depicted in Figure 2. The phenyl group in **A** can acquire either a contracted or an expanded conformation. Similarly to our previous research findings, it has been demonstrated that the destabilization of **OC** intermediates is correlated with the destabilization of transition states.^65^ Therefore, we investigated the initiation phase exclusively for the most stable conformers. Consequently, we suggest the criterion of Δ*G*_rel_ < 3 kcal/mol to select the intermediates that undergo a kinetically favored initiation phase, which in this study are all **OC**_A_ intermediates characterized by the expanded configuration of phenyl in **A**. This thermodynamic stabilization can be attributed to a more active alkene orientation, as described by Straub.^66^

**Figure 3:**
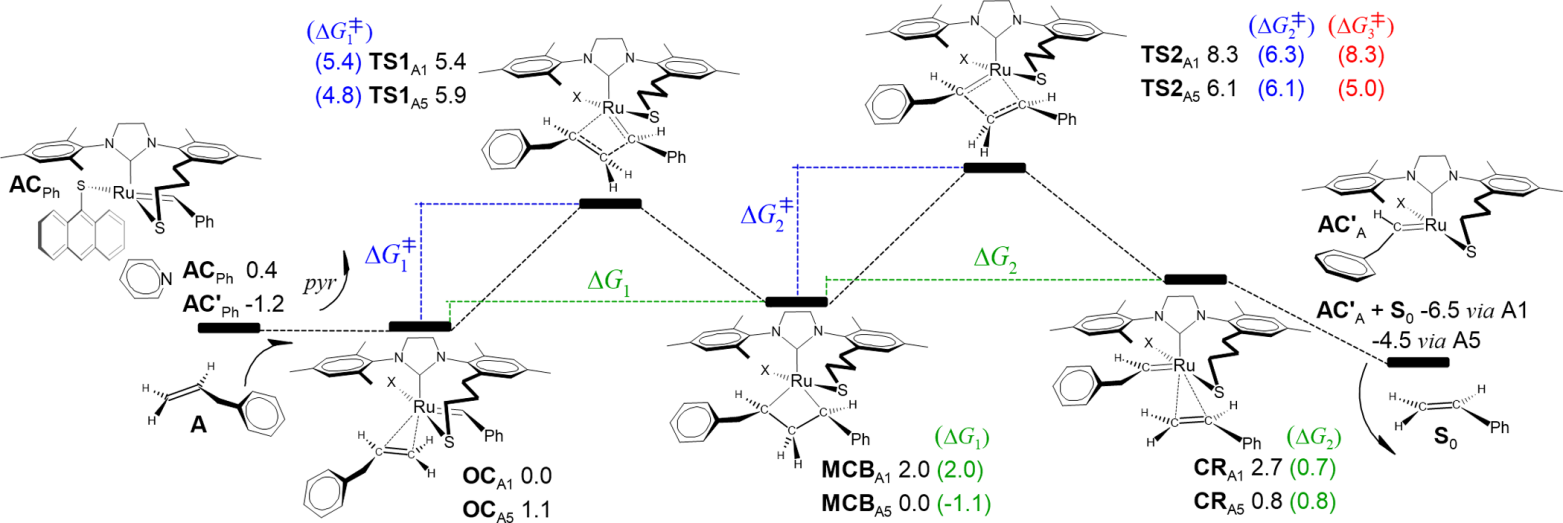
Gibbs free energy profiles for the initiation phase for the most stable **OC**_A_ species associated with productive metathesis. Illustration and energies (kcal/mol) are relative to **OC**_A1_. The equivalent schematization regarding the benzylidene of **AC′**_Ph_ localized in the opposite direction corresponds to path A5. Δ*G*_3_^‡^ = *G*(**TS2**) – *G*(**OC**).

The activated complex pyr/**AC**_Ph_ or pyr/**AC′**_Ph_ is isoenergetic with respect to **OC**_A_ since we did not observe significant energy variations for the exchange of pyridine with olefin **A** (Δ*G* = 0.4 and −1.2 kcal/mol respectively; see Figure 3). This fact indicates that catalyst activation is susceptible to reversibility when the concentration of **A** is insufficient. The 2,2-cycloaddition from **OC**_A_ via transition state **TS1**_A_ produces metallacyclobutane **MCB**_A_. The rupture of **MCB**_A_ through **TS2**_A_ leads to the 2,2-cycloreversion intermediate **CR**_A_, followed by the release of the propagating activated catalyst **AC**_A_ (or **AC′**_A_ in which alkylidene is *trans* to propyl-thiolate) and the corresponding olefin. Species **OC**_A1_ and **OC**_A5_ are associated with productive metathesis so that styrene (**S**_0_) is generated through the initiation route. On the other hand, **OC**_A3_ and **OC**_A7_ are related to nonproductive metathesis, so that substrate **S**_1_ (1-propene-1,3-diphenyl) is instead generated. The Gibbs free energy profiles, Δ*G*, for the initiation phase via **OC**_A1_ and **OC**_A5_ (productive metathesis) are depicted in Figure 3, along with the reaction energies and barriers for the 2,2-cycloaddition steps (Δ*G*_1_ and Δ*G*_1_^‡^) and 2,2-cycloreversion steps (Δ*G*_2_ and Δ*G*_2_^‡^).

To establish the kinetic bottleneck of the reaction, we evaluated all energy barriers in relation to the lowest-energy intermediate and highest-energy transition state, a concept that is referred as the energetic span model.^67−69^ That is, the third energy barrier reported in Figure 3 is calculated as Δ*G*_3_^‡^ = *G*(**TS2**_A_) – *G*(**OC**_A_) for each pathway. The Δ*G*_3_^‡^ value for path A1 is greater than the Δ*G*_1_^‡^ and Δ*G*_2_^‡^ values, indicating that the critical states of this reaction are **OC**_A1_ and **TS2**_A1_. In the case of path A5, Δ*G*_2_^‡^ is slightly greater than Δ*G*_3_^‡^; therefore, the crucial states of this pathway are **MCB**_A5_ and **TS2**_A5_ describing the 2,2-cycloreversion step. As indicated in Figure 3, our findings suggest that the catalyst can be promptly activated toward productive metathesis through pathways A1 and A5, which have overall costs of 8.3 and 6.1 kcal/mol, respectively. The generation of the active catalysts, **AC′**_A_ and **AC**_A_, through the release of styrene via paths A1 and A5, respectively, led to favorable driving forces of 6.5 and 4.5 kcal/mol, which were computed using the most stable isolated fragments: *G*(**AC′**_A_) or *G*(**AC**_A_) + *G*(styrene) – *G*(**OC**_A1_). In the cases of **AC′**_A_ and **AC**_A_, we optimized eight conformers and chose the most thermodynamically stable structures, as illustrated in Figure S3 in the Supporting Information. In contrast, nonproductive routes A3 and A7 are comparatively less kinetically competitive due to limiting initiation barriers higher than 10 kcal/mol. Furthermore, the formation of substrate **S**_1_ is endergonic, unlike the exergonic initiation through the release of styrene (see Figure S4 in the Supporting Information for a detailed description of the corresponding energy profiles).

As a final point, we employed the activation-strain model strategy^70−73^ to assess the relative energy of the system in the 2,2-cycloaddition transition states, considering the structural strain and intermolecular interactions involved. The strain energy quantifies the amount of energy required to alter the geometry of both olefin **A** and active catalyst **AC**_Ph_ (or **AC′**_Ph_) to form the structure of **TS1**_A_, while the interaction energy assesses the extent of interactions between the distorted fragments when they form **TS1**_A_. Similarly to our previous work,^64^ the overall cost Δ*G*_3_^‡^ for the initiation route is linearly correlated (*R*^2^ = 0.77) with the level of structural strain and, to some extent, the molecular interactions (*R*^2^ = 0.53, see inset in Figure 2). Consequently, faster reaction rates are associated with a lower degree of structural strain in the reacting entities as well as to moderate molecular interactions that prevent the formation of highly stabilized metallacyclobutanes, thereby promoting the course of the reaction.

### 3.2 The Propagation Route

**Figure 4:**
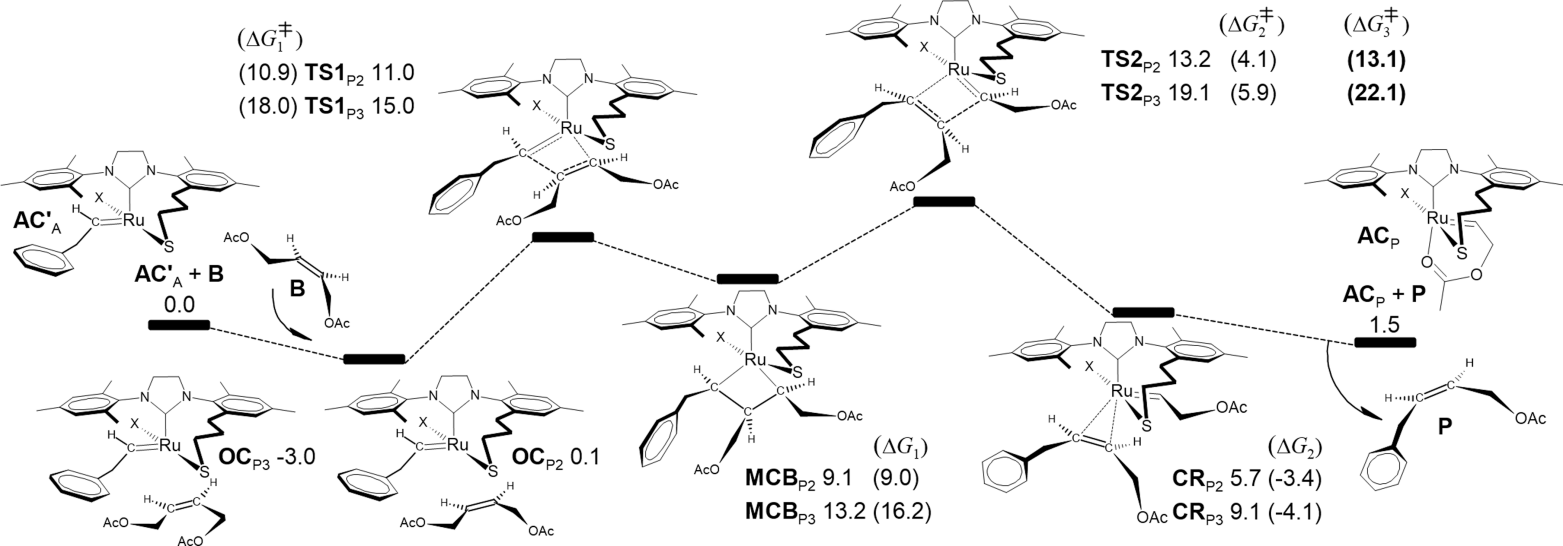
Gibbs free energy profiles (in kcal/mol) for the propagation phase through 2-butene-1,4-diyl diacetate as the substrate (**B**) and the catalyst activated via the metathesis of allylbenzene (**AC′**_A_).

In relation to the process of productive metathesis, the active catalysts **AC′**_A_ and **AC**_A_ are subsequently coordinated with compound **B** to form the propagating **OC**_P_ species. However, the flexibility of the structure of olefin **B** results in the formation of several conformers of **OC**_P_ (the selection of **OC**_P_ species is reported in Figure S5 in the Supporting Information). Similarly to the preceding subsection, our analysis of the propagation phase focused exclusively on the most stable conformers, specifically, **OC**_P2_ and **OC**_P3_. The corresponding reaction mechanisms are illustrated in Figure 4.

The Gibbs free energy profiles for the propagation phase through **OC**_P2,3_ are reported in relation to the sum of the energies of **AC′**_A_ and **B**. To obtain the energy of **B**, we selected the most stable structure from a group of three *cis* conformers (see Figure S3 in the Supporting Information for details). For example, the formation of **OC**_P2_ resulted in no energy change (Δ*G* = 0.1 kcal/mol) and the 2,2-cycloaddition energy barrier is Δ*G*_1_^‡^ = 10.9 kcal/mol. **MCB**_P2_ is destabilized since Δ*G*_1_ = 9.0 kcal/mol, but the 2,2-cycloreversion proceeds with an energy barrier of only Δ*G*_2_^‡^ = 4.1 kcal/mol under an exergonic process of Δ*G*_2_ = −3.4 kcal/mol. Olefin decoordination from **CR**_P2_ resulted in a favorable driving force of 4.2 kcal/mol, giving rise to the acetate propagating alkylidene (**AC**_P_) and the metathesis product **P**. The **OC**_P2_ and **TS2**_P2_ stationary points are the rate-limiting states in this part of the pathway, resulting in an overall cost of Δ*G*_3_^‡^ = 13.1 kcal/mol, so that the reaction can be carried out under laboratory conditions. In our prior communication,^64^ we reported equivalent energy barriers for a second-generation Grubbs catalyst, so that a similar catalytic activity is expected for the complex in question. Path P3 is kinetically less competitive but accessible since we calculated Δ*G*_3_^‡^ = 22.1 kcal/mol as the limiting barrier. Furthermore, if the phenyl-alkylidene is located in the opposite direction relative to **AC′**_A_ (that is, **AC**_A_ in which the phenyl-alkylidene is *cis* to propyl-thiolate), the propagating structure **OC**_P6_, which is analogous to **OC**_P2_, was destabilized by 6 kcal/mol compared to **OC**_P2_. Nonetheless, the overall energy required for the formation of **P** via path P6 was calculated to be only 9 kcal/mol, as illustrated in Figure S6 and discussed in the Supporting Information. Consequently, the catalyst in question is versatile, which means that metathesis products can be generated regardless of the orientation of the phenyl-alkylidene within the activated catalyst. Our calculations indicate that Z-stereoselective catalysts can be effectively designed by considering the bottom-bound mechanism, making our study a reference point for future synthetic procedures as an alternative to strategies based on the side-bound mechanism.

## 4 Conclusions

We performed predictive DFT calculations to assess the catalytic activity of a Ru-based complex containing an anthracene-9-thiolate ligand. The catalyst was designed in silico to induce the formation of the Z isomer of metathesis products through the bottom-bound mechanism. Additionally, the anthracene-9-thiolate ligand impedes the formation of the E isomer. Through an in-depth analysis of the thermodynamics and kinetics of the formulated mechanisms, we explored the potential applications of the Ru complex in the CM of allylbenzene and 2-butene-1,4-diyl diacetate. Our results indicate that this new catalyst should facilitate efficient catalyst activation, as the energy barrier for the initiation route was <15 kcal/mol essentially attributed to the Ru-pyridine bond dissociation. Furthermore, we demonstrated that the overall energy cost for olefin metathesis through the propagation phase, which leads to the synthesis of the Z isomer of the metathesis product, 4-phenyl-2-buten-1-yl acetate, was only 13 kcal/mol. Our findings hold significant promise for the future exploration of this new catalyst candidate through experimental studies, and we hope that they will serve as a useful guide for further computational investigations aimed at developing new Z-stereoselective catalysts. In this regard, we additionally proposed a laboratory procedure for the synthesis of this novel catalyst.

## Supporting Information Available

Discussion of alternative initiation and propagation phases; detailed analysis of molecular structures and energies of the investigated chemical species and their structural isomers, along with their respective DFT absolute energies (PDF)
Computed molecular Cartesian coordinates in a format convenient for visualization (XYZ)

## Supplementary Material

jp3c05021_si_001.pdf

jp3c05021_si_002.xyz
