# Supplementary material for: An Anthracene-Thiolate-Ligated Ruthenium Complex: Computational Insights into Z-Stereoselective Cross Metathesis
Source: J Phys Chem A. 2023 Nov 2;127(45):9465–72. doi: 10.1021/acs.jpca.3c05021 (PMC10658622; doi:10.1021/acs.jpca.3c05021)
Supplement: Supplementary file 1 — jp3c05021_si_001.pdf [file jp3c05021_si_001.pdf]

Supporting Information

An Anthracene-Thiolate-Ligated Ruthenium Complex: Computational Insights into Z-Stereoselective Cross Metathesis

J. Pablo Martínez[§]*, Bartosz Trzaskowski[§]*

orcid.org/0000-0002-6589-790X; email: p.martinez@cent.uw.edu.pl

orcid.org/0000-0003-2385-1476; email: b.trzaskowski@cent.uw.edu.pl

[§] Centre of New Technologies, University of Warsaw, 02-097 Warszawa, Poland

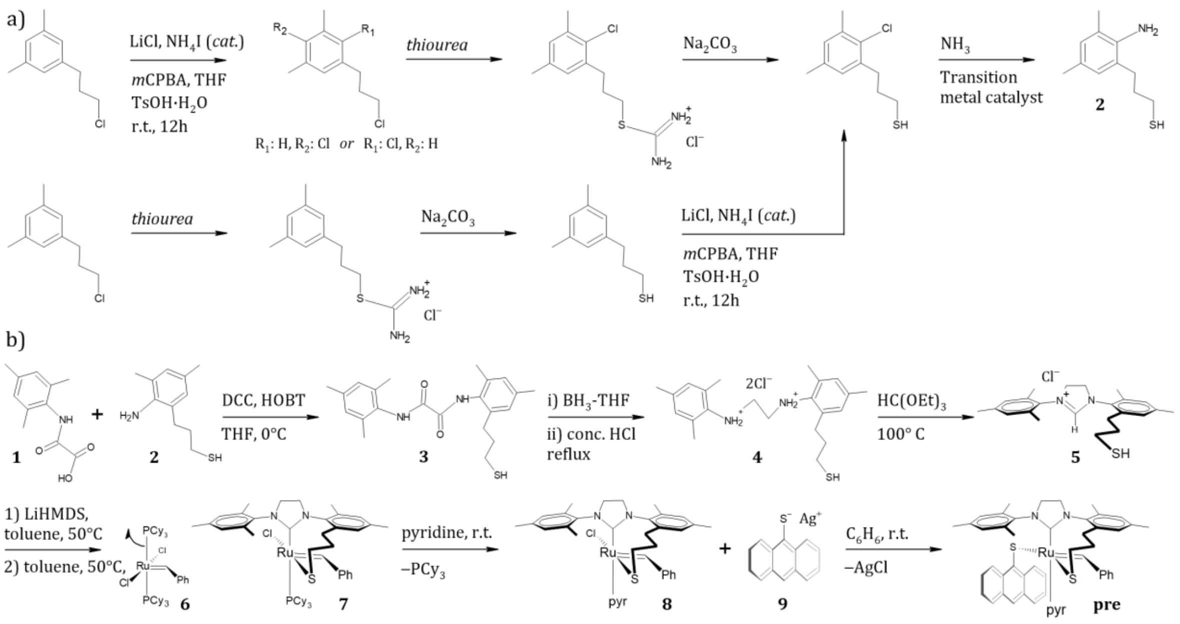

Figure S1. a) Two procedures proposed for the synthesis of the thiol aniline **2**. *m*CPBA: *m*-chloroperbenzoic acid, TsOH·H_2O: *p*-toluenesulfonic acid. b) Proposed synthesis of the thiol NHC ligand **5**, along with the preparation of the precatalyst studied in this work through modifications of Grubbs 1st generation complex **6**. DCC: 1,3-dicyclohexylcarbodiimide, HOBT: 1-hydroxybenzotriazole.

The synthesis of thioether-imidazolium chlorides was previously reported by Shirai et al.[†] To this end, we have adapted their method, which is presented in Figure S1 (species **1** to **5**). Aniline **2** is prepared using traditional techniques. Successively, the Grubbs first-generation catalyst (**6**) is utilized to attach the NHC to the Ru center (**7**), and the phosphine ligand is substituted by a pyridine unit (**8**). Subsequent preparation of the precatalyst (**pre**) is inspired by the method developed by Grubbs et al.[††] Similar to the synthesis of silver sulfonates and phosphates, the silver anthracene-9-thiolate (**9**) should be synthesized by reacting anthracene-9-thiol and $AgNO_3$ in the presence of Na_2CO_3. It is expected that a standard ligand exchange reaction between chloride in **8** and silver thiolate **9** will produce **pre**, with AgCl precipitating out. In the case of the catalysts prepared by Grubbs et al.,[††] chloride was mono- (>75%) and di-substituted (<20%) by sulfonates or phosphates. In this work, the propyl-thiolate of the NHC used in the preparation of **7** ensures posterior mono-substitutions with **9** in the synthesis of **pre**.

[†] Kuriyama, M.; Shimazawa, R.; Shirai, R. *Tetrahedron* **2007**, 63, 9393–9400.

[††] Teo, P.; Grubbs, R. H. *Organometallics* **2010**, 29, 6045–6050.

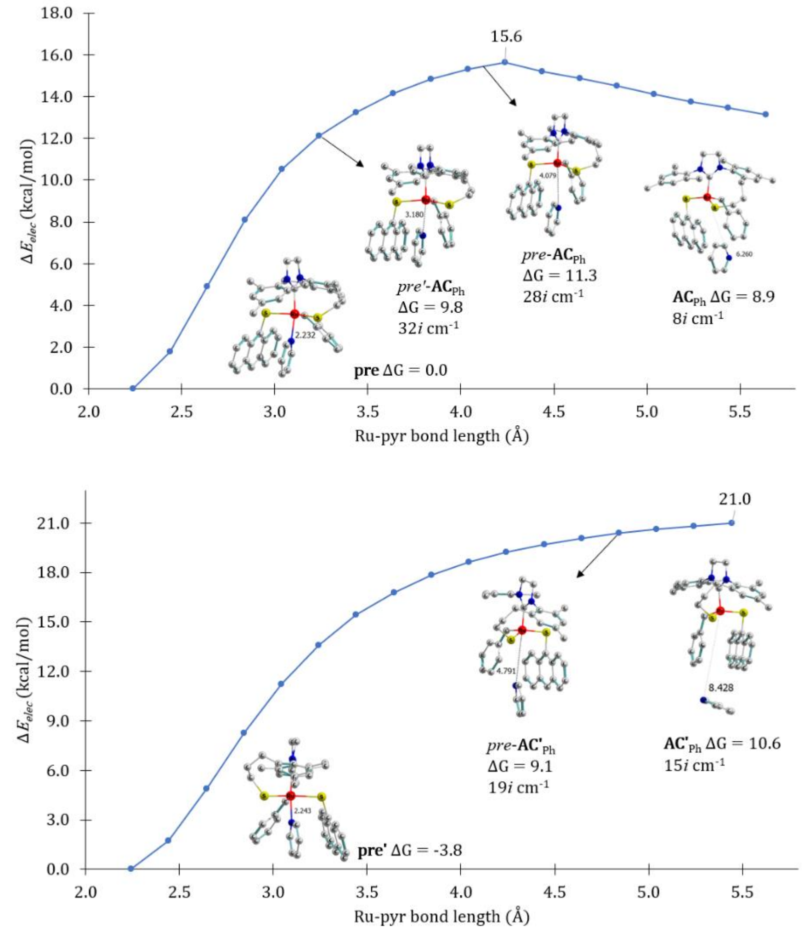

Figure S2. Potential energy surface evaluated for the Ru-pyridine bond dissociation in the catalyst under investigation.

a) Reactants and metathesis (sub)products.

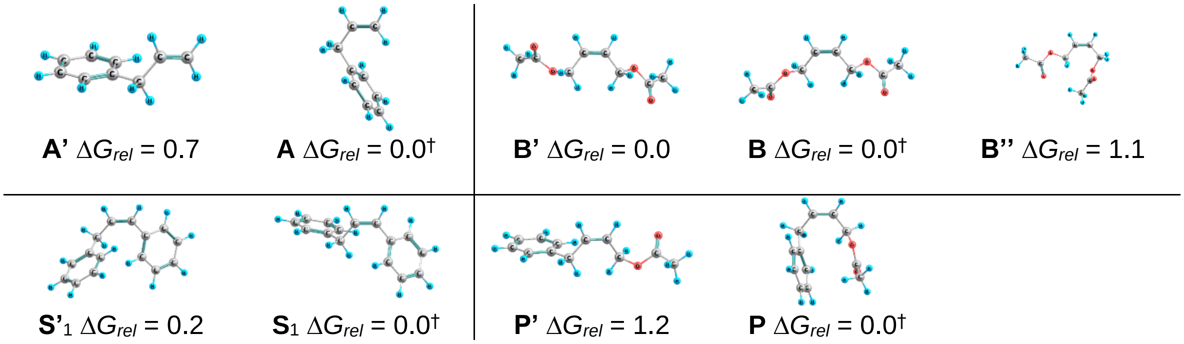

b) Activated catalyst generated through the initiation route.

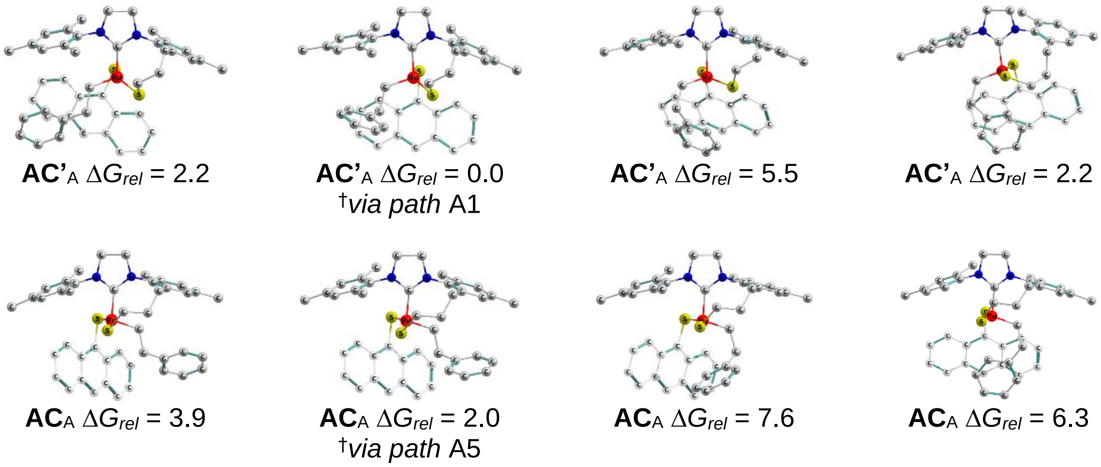

c) Activated catalyst **AC** and propagating catalyst **AC**$_P$.

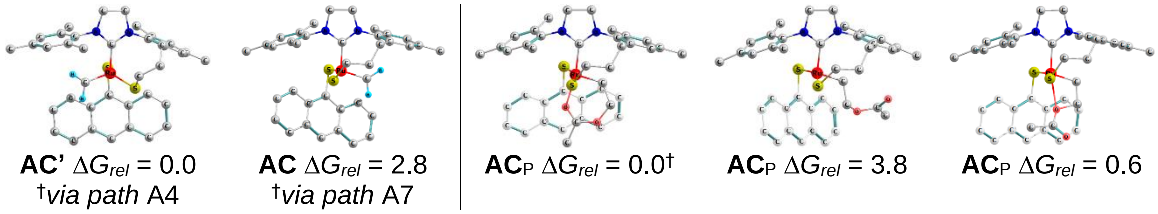

Figure S3. Structural and energy comparisons of conformers of reactants, activated catalysts, and products related to the initiation and propagation routes. †Selected for further analysis.

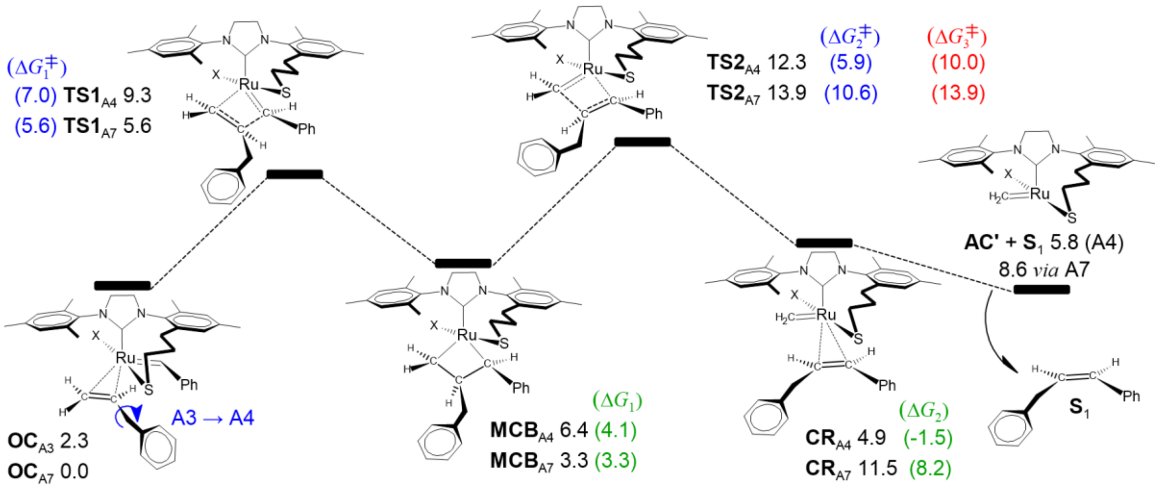

Figure S4. Gibbs free energy profiles for the initiation phase for the most stable **OC**$_A$ species associated with nonproductive metathesis. The illustration corresponds to path A3 that was spontaneously transformed into A4 during the search of **TS1**$_{A3}$ through the respective linear-transit calculation. Energies (kcal/mol) are relative to **OC**$_{A1}$.

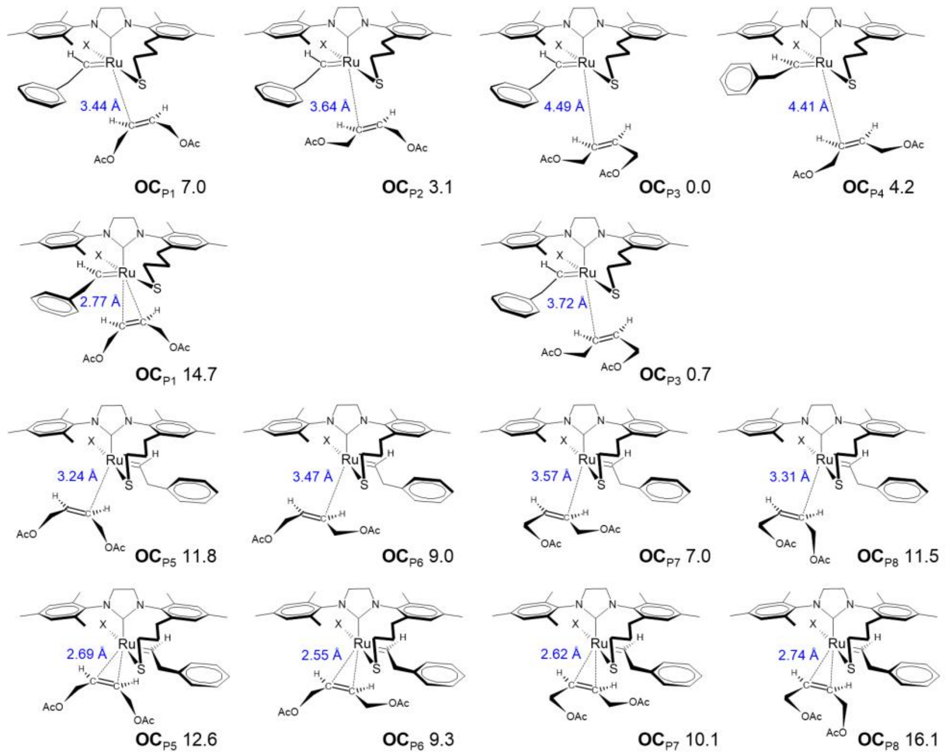

Figure S5. Conformers of the catalyst activated with allylbenzene (**AC**$_A$) coordinated to 2-butene-1,4-diyl diacetate related to the propagation route (**OC**$_P$), and Gibbs free energy comparisons in kcal/mol relative to **OC**$_{P3}$.

Conformers **OC**$_{P}$ describing the propagation phase of productive metathesis exhibited the olefin **B** located at a distant position ($d_{Ru\text{-}C}$ > 3 Å) with respect to the activated catalyst **AC**$_{A}$. Linear-transit calculations led to **OC**$_{P}$ species characterized by $d_{Ru\text{-}C}$ < 3 Å, but in some cases (P1, P7, and P8) the conformers were destabilized, or it was not even possible to optimize the respective **OC**$_{P}$ species (P2 and P4). Such a result suggests that the interactions between the olefin and the active catalyst may be hindered to some extent. However, the thermodynamic stabilization of conformers **OC**$_{P2,3}$ is comparable with that of the isolated olefin **B** and activated catalyst **AC**$_{A}$, and productive metathesis is indeed possible via paths P2 and P3 as shown in the main manuscript.

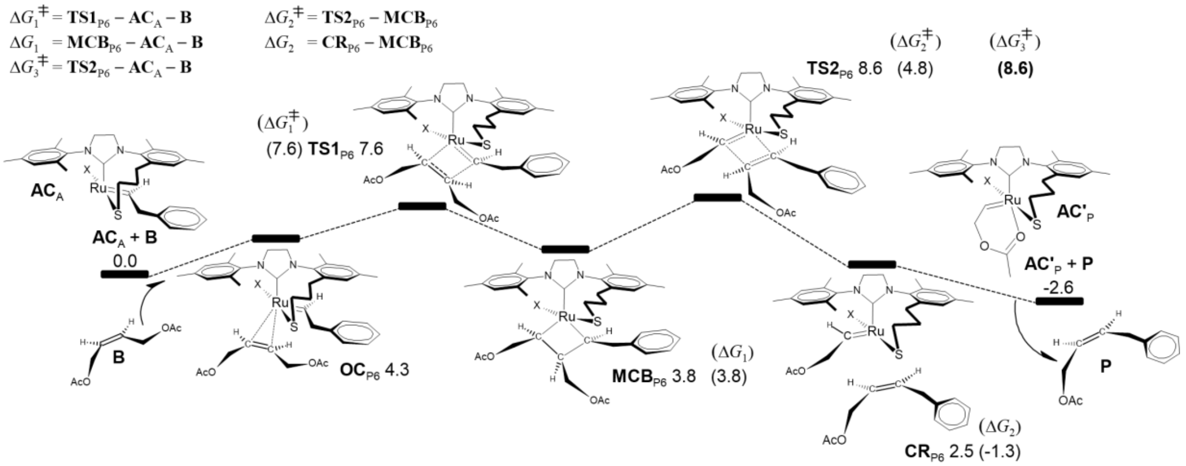

Figure S6. Gibbs free energy profile (kcal/mol) for the propagation phase through species **OC**$_{P6}$ associated with productive metathesis.

In the main manuscript it was shown that the initiation routes via **OC**$_{A1}$ and **OC**$_{A5}$ are possible so that the active catalysts **AC'**$_{A}$ and **AC**$_{A}$ are respectively generated, mainly differentiated by the orientation of the phenyl-alkylidene. Species **OC**$_{Pn}$ (n = 1 to 4) react with **AC'**$_{A}$, but **OC**$_{Pn}$ (n = 5 to 8) which coordinate with **AC**$_{A}$ are destabilized by at least 7 kcal/mol as reported in Figure S5. The results illustrated in Figure S6 solves the question whether the propagation route exclusively occurs at one side of the catalyst (via **OC**$_{P2}$ or **OC**$_{P3}$), which demonstrate that productive metathesis is also feasible via **OC**$_{P6}$ (analogous to **OC**$_{P2}$) at an overall cost of 8.6 kcal/mol.

Table S1. Gas-phase electronic energy (E_{elec}) and solvation (E_{PCM}) in CH_2Cl_2 in a.u. at M06(D3)/6-311G++**~LACV3P++**. Zero-point energy (*ZPE*) and thermal correction to enthalpy (H_{tot}) in kcal mol^{-1}, and entropy (S_{tot}) in cal mol^{-1} K^{-1} at gas-phase B3LYP(D3)/6-31G**~LACVP**. Gibbs energies G_{solv} in a.u. as defined in the main manuscript.

Initiation routes for productive and non-productive metathesis

structure	*ifreq* (cm^{-1})	E_{elec}	E_{PCM}	*ZPE*	S_{tot}	H_{tot}	G_{solv}
**pre**	-	-2950.2647	-0.0208	540.42	265.2	31.88	-2949.4994
pre'-**AC**$_{Ph}$	32.3*i*	-2950.2462	-0.0225	539.67	264.6	31.69	-2949.4839
pre-**AC**$_{Ph}$	28.4*i*	-2950.2414	-0.0235	539.24	266.9	31.88	-2949.4814
**AC**$_{Ph}$	7.9*i*	-2950.2464	-0.0226	539.45	266.8	31.87	-2949.4852
**pre'**	-	-2950.2705	-0.0216	540.52	264.2	31.78	-2949.5055
pre-**AC'**$_{Ph}$	18.6*i*	-2950.2425	-0.0254	539.20	267.5	31.92	-2949.4848
**AC'**$_{Ph}$	14.9*i*	-2950.2395	-0.0258	539.05	267.8	31.92	-2949.4826
**OC**$_{A1}$	-	-3050.8559	-0.0213	586.94	279.9	34.11	-3050.0204
**OC**$_{A2}$	-	-3050.8468	-0.0207	586.91	279.7	34.10	-3050.0107
**OC**$_{A3}$	-	-3050.8514	-0.0225	586.36	275.3	33.56	-3050.0167
**OC**$_{A4}$	-	-3050.8455	-0.0219	586.32	279.5	34.10	-3050.0114
**OC**$_{A5}$	-	-3050.8545	-0.0217	587.11	278.3	33.94	-3050.0186
**OC**$_{A6}$	-	-3050.8513	-0.0206	587.10	277.3	33.86	-3050.0139
**OC**$_{A7}$	-	-3050.8551	-0.0223	586.76	278.1	33.97	-3050.0203
**OC**$_{A8}$	-	-3050.8476	-0.0222	586.43	279.5	34.09	-3050.0137
**TS1**$_{A1}$	218.6*i*	-3050.8486	-0.0218	587.08	273.7	33.33	-3050.0117
**MCB**$_{A1}$	1.8*i*	-3050.8550	-0.0229	587.83	270.7	32.98	-3050.0172
**TS2**$_{A1}$	115.3*i* †	-3050.8435	-0.0223	586.50	271.0	32.98	-3050.0072
**CR**$_{A1}$	-	-3050.8487	-0.0233	586.54	280.6	34.16	-3050.0161
**TS1**$_{A3}$	179.9*i*	-3050.8423	-0.0218	586.98	273.7	33.31	-3050.0056
**MCB**$_{A3}$	-	-3050.8475	-0.0226	587.91	274.0	33.40	-3050.0101
**TS2**$_{A3}$	198.4*i*	-3050.8347	-0.0243	586.81	273.9	33.33	-3050.0008
**CR**$_{A3}$	12.4*i*	-3050.8452	-0.0229	585.67	276.5	33.64	-3050.0125
**TS1**$_{A5}$	209.7*i*	-3050.8468	-0.0223	586.90	274.7	33.42	-3050.0110
**MCB**$_{A5}$	-	-3050.8570	-0.0229	587.89	275.3	33.53	-3050.0204
**TS2**$_{A5}$	211.7*i* †	-3050.8455	-0.0236	586.47	270.9	32.96	-3050.0107
**CR**$_{A5}$	-	-3050.8535	-0.0223	586.76	279.5	34.07	-3050.0191
**TS1**$_{A7}$	287.0*i*	-3050.8474	-0.0226	587.08	273.6	33.29	-3050.0114
**MCB**$_{A7}$	-	-3050.8517	-0.0232	587.89	274.4	33.44	-3050.0151
**TS2**$_{A7}$	247.2*i*	-3050.8323	-0.0240	586.83	274.5	33.40	-3049.9983
**CR**$_{A7}$	-	-3050.8324	-0.0244	585.89	280.8	34.26	-3050.0020

† Additional imaginary frequencies 2.8*i* and 7.5*i* cm^{-1} were ignored in these cases, respectively. Even though we tried to remove small imaginary frequencies, in these cases were ignored after several attempts.

structure	*ifreq* (cm^{-1})	E_{elec}	E_{PCM}	*ZPE*	S_{tot}	H_{tot}	G_{solv}
Reactants and (sub)products †							
**A'**	-	-348.7590	-0.0039	101.84	87.5	5.55	-348.6333
**A**	-	-348.7599	-0.0042	101.82	87.4	5.55	-348.6345
**B'**	-	-612.7754	-0.0136	122.73	114.9	8.91	-612.6338
**B**	-	-612.7745	-0.0142	122.61	115.4	8.96	-612.6339
**B''**	-	-612.7767	-0.0117	122.88	112.6	8.74	-612.6321
**S**$_0$	-	-309.4712	-0.0043	83.99	80.8	4.76	-309.3724
**S**$_1$	-	-579.6888	-0.0063	153.57	107.7	8.08	-579.4887
**S'**$_1$	-	-579.6896	-0.0059	153.76	106.7	8.00	-579.4884
**P**	-	-615.8794	-0.0099	147.18	112.5	8.82	-615.6942
**P'**	-	-615.8768	-0.0102	147.25	113.6	8.82	-615.6923
pyr	-	-248.1547	-0.0061	55.80	67.3	3.27	-248.0987
Catalyst activated through initiation routes							
**AC'**$_A$	2.7*i*	-2741.3589	-0.0220	500.02	246.3	28.98	-2740.6548
**AC'**$_A$	-	-2741.3636	-0.0205	500.41	249.8	29.41	-2740.6584
**AC'**$_A$	-	-2741.3542	-0.0204	500.13	250.5	29.49	-2740.6495
**AC'**$_A$	-	-2741.3588	-0.0202	499.63	251.1	29.59	-2740.6549
**AC**$_A$	-	-2741.3572	-0.0207	500.29	249.4	29.43	-2740.6521
**AC**$_A$	-	-2741.3605	-0.0201	500.34	250.5	29.50	-2740.6552
**AC**$_A$	-	-2741.3507	-0.0209	500.29	250.1	29.46	-2740.6462
**AC**$_A$	-	-2741.3527	-0.0204	499.85	249.8	29.47	-2740.6483
**AC'**	-	-2471.1221	-0.0202	430.63	226.8	25.96	-2470.5224
**AC**	-	-2471.1188	-0.0190	430.47	226.3	25.97	-2470.5179
Structures **OC**$_P$ *characterized by* Ru-C$_{olefin}$ > 3 Å *related to the propagation phase*							
**OC**$_{P1}$	-	-3354.1696	-0.0270	625.37	308.6	38.20	-3353.2857
**OC**$_{P2}$	-	-3354.1754	-0.0267	625.03	309.7	38.35	-3353.2920
**OC**$_{P3}$	3.8*i*	-3354.1807	-0.0268	624.71	306.1	37.90	-3353.2970
**OC**$_{P4}$	-	-3354.1681	-0.0311	624.54	310.9	38.46	-3353.2904
**OC**$_{P5}$	-	-3354.1622	-0.0265	625.11	309.0	38.27	-3353.2782
**OC**$_{P6}$	-	-3354.1654	-0.0266	624.63	310.0	38.42	-3353.2826
**OC**$_{P7}$	-	-3354.1685	-0.0270	624.66	309.3	38.37	-3353.2858
**OC**$_{P8}$	-	-3354.1621	-0.0269	624.96	308.9	38.29	-3353.2787
Structures **OC**$_P$ *characterized by* Ru-C$_{olefin}$ < 3 Å *related to the propagation phase* †							
**OC**$_{P1}$	-	-3354.1575	-0.0271	625.27	308.2	38.20	-3353.2736
**OC**$_{P3}$	-	-3354.1804	-0.0251	624.80	309.9	38.38	-3353.2958
**OC**$_{P5}$	-	-3354.1588	-0.0292	625.30	307.8	38.19	-3353.2768
**OC**$_{P6}$	-	-3354.1693	-0.0250	625.27	305.0	37.96	-3353.2822
**OC**$_{P7}$	-	-3354.1650	-0.0267	624.78	306.7	38.17	-3353.2809
**OC**$_{P8}$	-	-3354.1559	-0.0263	625.21	308.6	38.30	-3353.2713

† The Cartesian coordinates corresponding to these sets of structures were omitted from the attached .xyz file.

Propagation routes for productive metathesis

structure	*ifreq* (cm^{-1})	E_{elec}	E_{PCM}	*ZPE*	S_{tot}	H_{tot}	G_{solv}
**TS1**$_{P2}$	148.1*i*	-3354.1622	-0.0269	626.06	300.6	37.32	-3353.2747
**MCB**$_{P2}$	1.5*i*	-3354.1672	-0.0273	627.08	298.0	37.01	-3353.2777
**TS2**$_{P2}$	141.2*i*	-3354.1575	-0.0267	625.65	302.8	37.53	-3353.2711
**CR**$_{P2}$	-	-3354.1692	-0.0255	625.38	306.8	38.10	-3353.2831
**TS1**$_{P3}$	143.0*i*	-3354.1565	-0.0251	625.51	301.2	37.41	-3353.2683
**MCB**$_{P3}$	-	-3354.1609	-0.0259	626.81	301.2	37.46	-3353.2713
**TS2**$_{P3}$	146.1*i*	-3354.1493	-0.0258	625.56	301.6	37.44	-3353.2617
**CR**$_{P3}$	-	-3354.1636	-0.0260	625.41	306.2	38.01	-3353.2778
**TS1**$_{P6}$	126.2*i*	-3354.1654	-0.0256	625.84	300.7	37.34	-3353.2769
**MCB**$_{P6}$	-	-3354.1730	-0.0259	627.14	301.5	37.46	-3353.2830
**TS2**$_{P6}$	131.6*i*	-3354.1640	-0.0255	626.08	301.3	37.36	-3353.2754
**CR**$_{P6}$	-	-3354.1693	-0.0270	625.36	307.6	38.13	-3353.2850
Propagating catalyst							
**AC**$_{P}$	-	-2738.2610	-0.0217	475.98	248.7	29.24	-2737.5956
**AC**$_{P}$	-	-2738.2550	-0.0215	476.06	249.6	29.35	-2737.5896
**AC**$_{P}$	-	-2738.2602	-0.0213	475.97	249.0	29.27	-2737.5946
**AC’**$_{P}$	-	-2738.2644	-0.0229	476.47	246.8	29.05	-2737.5989

The structures reported in the attached XYZ file that contains Cartesians coordinates follow the same sequence as reported in Table S1.
